# Targeting human progesterone receptor (PR), through pharmacophore-based screening and molecular simulation revealed potent inhibitors against breast cancer

**DOI:** 10.1038/s41598-024-55321-0

**Published:** 2024-03-21

**Authors:** Muhammad Shahab, Peng Ziyu, Muhammad Waqas, Guojun Zheng, Yousef A. Bin Jardan, Gezahign Fentahun Wondmie, Mohammed Bouhrhia

**Affiliations:** 1 State Key Laboratories of Chemical Resources Engineering, Beijing University of Chemical Technology, Beijing, 100029 People’s Republic of China; 2 School of chemistry and chemical engineering, Wuhan University of Science and Technology, Wuhan, 430081 People’s Republic of China; 3 Natural and Medical Sciences Research Center, University of Nizwa, Birkat Al-Mouz, 616 Nizwa, Oman; 4 Department of Pharmaceutics, College of Pharmacy, King Saud University, P. O. BOX 2455, 11451 Riyadh, Saudi Arabia; 5 Department of Biology, Bahir Dar University, P.O. Box 79, Bahir Dar, Ethiopia; 6 Laboratory of Biotechnology and Natural Resources Valorization, Faculty of Sciences, Ibn Zohr University, 80060 Agadir, Morocco

**Keywords:** HPR, Breast cancer, Molecular docking, MD simulation, MMBPSA, Biochemistry, Cancer

## Abstract

Breast cancer, the prevailing malignant tumor among women, is linked to progesterone and its receptor (PR) in both tumorigenesis and treatment responsiveness. Despite thorough investigation, the precise molecular mechanisms of progesterone in breast cancer remain unclear. The human progesterone receptor (PR) serves as an essential therapeutic target for breast cancer treatment, warranting the rapid design of small molecule therapeutics that can effectively inhibit HPR. By employing cutting-edge computational techniques like molecular screening, simulation, and free energy calculation, the process of identifying potential lead molecules from natural products has been significantly expedited. In this study, we employed pharmacophore-based virtual screening and molecular simulations to identify natural product-based inhibitors of human progesterone receptor (PR) in breast cancer treatment. High-throughput molecular screening of traditional Chinese medicine (TCM) and zinc databases was performed, leading to the identification of potential lead compounds. The analysis of binding modes for the top five compounds from both database provides valuable structural insights into the inhibition of HPR for breast cancer treatment. The top five hits exhibited enhanced stability and compactness compared to the reference compound. In conclusion, our study provides valuable insights for identifying and refining lead compounds as HPR inhibitors.

## Introduction

Breast cancer (BC) is a deadly malignancy that profoundly affects the lives of millions of women and their families worldwide^1^. It stands as the second most prevalent cause of cancer-related fatalities among women. To date, breast cancer is currently the most common form of malignant tumor affecting women globally^2^. Progesterone and the nuclear progesterone receptor have a critical role in controlling mammary gland tumorigenesis^3,4^. In breast cancer, the estrogen receptor (ER) and the progesterone receptor (PgR) play a vital role in determining the responsiveness to endocrine therapies^5^. In breast cancer, relying solely on the expression of ER and PgR is unlikely to determine the most suitable treatment approach for a patient. Instead, multifactorial techniques are required to analyze the expression of tumor biomarkers for making informed decisions regarding the optimal treatment regimen^6^. Progestogen defines the category of hormone molecules that act like progesterone in the uterus^7^. Progesterone plays a crucial role in regulating cell proliferation and differentiation in female reproductive processes^8^. The term "progestin" is commonly used to encompass both natural progesterone and a range of synthetic hormone molecules. The human progesterone receptor (HPR) functions as a ligand-induced transcription factor (TF), directly interacting with specific progesterone response elements (PREs) located in the promoter region of target genes^9^. Apart from its function as a transcription factor, the Human progesterone receptor (HPR) can activate signal transduction pathways through rapid or non-genomic mechanisms, leading to cellular responses^10^. Progestin induces the expression of certain genes that are involved in promoting breast cancer growth. Interestingly, these genes do not contain progesterone response elements (PREs) in their promoters, which are typical binding sites for progesterone receptor (PR) to directly regulate gene expression. A remarkable point is that Breast cancer is a clinically heterogeneous disease, associated with a large number of gene mutations. Detection of the mutated genes clarifies the genetic and molecular mechanisms of the disease; furthermore, it significantly increases the chances of finding a successful treatment^11^. In other words, recent findings on the molecular mechanisms of Breast cancer could lead to the discovery of new drug candidates^12^. When ligands bind to HPR, a conformational change is induced, activating HPR to bind to DNA and regulate transcription. Despite extensive research, the precise molecular mechanisms governing the action of progesterone in breast cancer remain incompletely understood^13^. While it is known that progesterone plays a role in breast cancer growth and development, the specific molecular pathways and interactions involved in this process have not been completely elucidated. Mifepristone (RU-486) is a well-known progesterone inhibitor that is primarily used for medical abortion and emergency contraception. It is also being studied for its potential in treating certain hormone-related conditions, including breast cancer^14^, and Ulipristal Acetate is another progesterone receptor modulator used for emergency contraception and the management of uterine fibroids^15^. The Existing progesterone inhibitors may have side effects that limit their use. These can include nausea, vomiting, and changes in menstrual bleeding patterns. For this purpose developing therapies with greater selectivity for specific tissues can help reduce side effects and improve the overall safety profile. Given the vital role of the Human progesterone receptor (HPR) in breast cancer, it represents a critical therapeutic target for the treatment of breast cancer^16^. Thus, there is an imperative to expedite the design of small molecule therapeutics or drugs that can efficiently inhibit HPR. Throughout history, natural products have been utilized as natural remedies for the treatment of various diseases since the early identification of disease^17^. The identification of these molecules is accelerated by employing state-of-the-art computational methods, which encompass molecular screening, simulation, and free energy calculation techniques^18^. Keeping in view the pharmacological potential of natural products and the speed and accuracy, this study utilizes Pharmacophore-based virtual screening and molecular simulation to target the human progesterone receptor (HPR) in breast cancer treatment^19–21^. High throughput molecular screening was conducted using Traditional Chinese Medicine (TCM) and zinc databases, leading to the identification of potential lead molecules. The top two compounds from each database were analyzed for their binding modes. As a result, this study offers a structural basis for the inhibition of HPR in breast cancer treatment.

## Methodology

**Figure 1:**
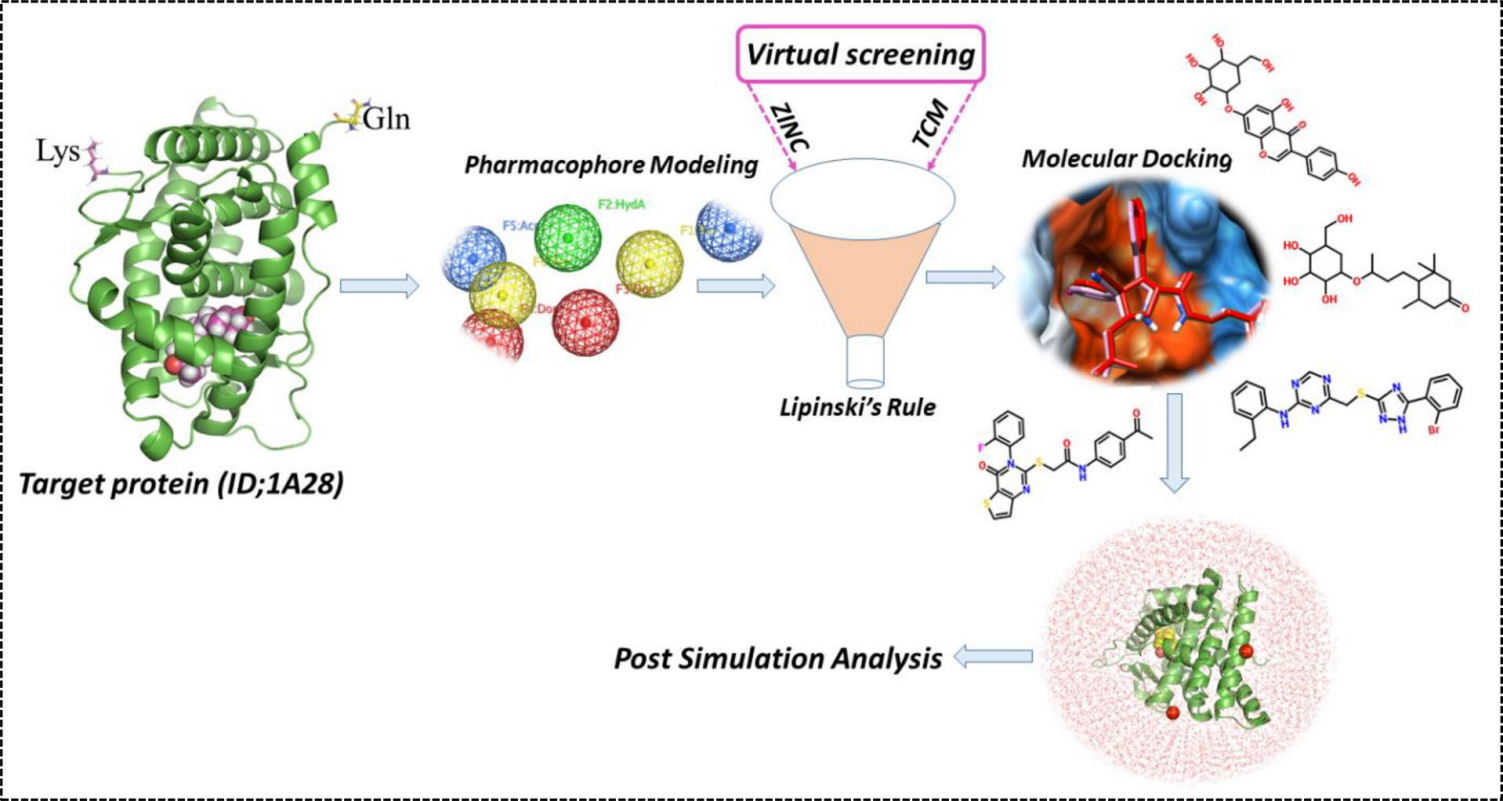
Workflow and tools used in this study for designing of potent lead drugs candidates. (Pro Lab v2018; https://originpro.informer.com/8.5/).

The overall mechanism and various tools used in this study for designing lead compound by rational drug design are depicted in Fig. 1.

### Retrieval of the structure

In order to perform the docking studies, the 3-dimensional structure of proteins is one of the necessary requirements. Therefore, the protein data bank (PDB) database was used to download the structures of Human progesterone hormone (PBD ID: 1A28, Resolution: 1.80 Å and AA: 256)^22^. The ligand (progesterone) was taken as a control and residues interacting with the control compound were taken as active site residues. Following the structure retrieval, they were imported into the interface of Maestro and were subjected to an optimization pipeline using the LigPrep module of the same software. Noteworthy, the OPLS3 force field for the structure optimization. Missing hydrogen atoms were also incorporated at the standard protonation state pH 7^23,24^. Docking studies were performed using AutoDock vina docking algorithms, and virtual screening was conducted using compound databases^25^. Based on the position occupied by the identified active site residues, a grid box of size x = 15.5161166153 Å, y = 20.48523316176 Å, and z = 19.5651016016 Å, with a center dimension of x = 13.208380341, y = 49.523715066, and z =− 30.7726860376 were set to define the active site. The docking results were analyzed, including binding affinities and interaction visualizations^26^.

### Pharmacophore model generation & validation

To find the lead compound against Human progesterone receptor (HPR) Pharmacophore based virtual screening were created based on the protein complex with the ligand. The selected protein was analyzed using pharmacophore tool in Molecular operating environment (MOE), for H-bond donors/acceptors, hydrophilicity, lipophilic features and ionizable charges^27–29^. Seven best pharmacophore features, that is, three hydrogen bond acceptor (HBA), two hydrophobic, and, two aromatic features were prioritized for generation of pharmacophore model. The model was then validated by two methods; a test datasets called in-house dataset. With the help of the research of our collaborators, the three-dimensional structures of 1,600 compounds were put into a database called an In-house. These compounds have a wide range of structures, with many different core scaffolds and substitution patterns. Second set of thirty nine active compound were taken from the literature study and then screened against the generated pharmacophore model^30^. This was used to assess the accuracy of our predicted model through protein–ligand interactions.

### Pharmacophore-based screening of natural product compound libraries

The validated pharmacophore model was applied to search databases or compound libraries (TCM and ZINC) for potential hits or new molecules that matched the pharmacophores features^31,32^. Based on docking interactions and score, the complex structure of most potential inhibitor was employed for the generation of ligand-based pharmacophore model accessed through MOE. Based on the screening, structurally diverse hits were recovered presenting a better fit to the generated pharmacophore model. Compounds with molecular weights > 500KD; H-bond donor > 5; H-bond Acceptor > 10; and Logp o/w > 5 were selected.

### Molecular interaction study and selection of lead compounds

Molecular docking is a computational technique used to predict the preferred binding mode and binding affinity of a small molecule (ligand) to a macromolecular target (receptor)^33^. In the context of drug discovery and development, molecular docking plays a crucial role in identifying potential lead compounds that can interact with a specific target protein, such as a receptor involved in a disease process^34^. The molecular docking typically involves the several steps; (1) Preparation the 3D structure of the target receptor obtained from experimental data. This involves adding missing atoms, assigning charges, and optimizing the protein structure, (2) Define a 3D grid around the active site or binding pocket of the target receptor, where ligands are expected to bind, (3) Preparation of the small molecule ligands (e.g. natural product compounds) by optimizing their geometry, adding hydrogen atoms, and assigning partial charges. Finally, the top-scoring complexes were then run through a molecular dynamics simulation using Amber v22 package.

### Molecular simulation

A molecular dynamic (MD) simulation was performed to investigate the dynamic behavior of proteins upon inhibitor binding at the atomic level. MD simulations were performed on the docked conformations of the chosen hits inside the HPR active pocket. A solvent box (OPC) optimal point charge was added around each system, and ions were added to neutralize the charge. Next, each system underwent energy minimization using a minimization algorithm such as steepest descent and conjugate gradient. The minimization process continued until the system reached a convergence criterion, such as a maximum force or energy change threshold. To allow each system to reach the desired simulation temperature and equilibrate, a temperature coupling algorithm (such as Langevin Dynamics or Berendsen thermostat) was used to gradually heat the system from a low temperature. Long-range electrostatic interactions were calculated using the Particle Mesh Ewald (PME) method, while van der Waals forces were calculated using Lennard-Jone's potential^35^. Each system was equilibrated at the target temperature and pressure for a certain period of time in several stages, including positional restraint, slow heating, and equilibration without restraints. To maintain covalent bond lengths, the SHAKE algorithm was used to constrain bond lengths and angles. The pressure of the system was controlled using a barostat such as Berendsen or Andersen^36^. After equilibration, each system was simulated for a production time of 1000 ns using a molecular dynamics algorithm such as NPT or NVT ensemble^37^. In this step, simulation parameters including time step and cutoff distances were set. Finally, the trajectory obtained from the production simulation was analyzed using CPPTRAJ or PTRAJ modules^38^. For Structural visualization, and graphical representation, PyMol v1.7, and Origin Pro Lab v2018, were used^39,40^.

#### Post trajectory analysis

In the post-trajectory analysis phase of our study, following molecular dynamics (MD) simulation, we conducted several analyses to investigate the behavior of the reference/PR complex and the lead hits. Root mean square deviation (RMSD) analysis provided insights into the stability and structural changes of the complex over time. Root mean square fluctuation (RMSF) analysis revealed the flexibility and local fluctuations of PR residues. Hydrogen Bonding (HB) analysis identified key residues involved in stabilizing the complex. Radius of Gyration (RoG) analysis assessed the compactness and overall shape of the complex^41^. These comprehensive post-trajectory analyses provided valuable information about the dynamics, flexibility, intermolecular interactions, and overall behavior of the HPR-progesterone complex, contributing to a deeper understanding of its structural dynamics.

### Binding free energy estimation through MM/GBSA analysis

Insights into the process of how a protein identifies its biologically significant ligand or a small molecule inhibitor significantly impact the discovery of effective small molecule treatments. This approach has the advantage over others as it is less time-consuming and computationally inexpensive^42,43^. It has been widely used to determine the BFE for protein–protein and protein–ligand complexes.

The following equation was used to calculate each term in the total binding energy.

1 $${\Delta G}_{bind}= {G}_{(complex,\, solvated)}- {G}_{\left(protein,\, solvated\right)}- {G}_{\left(ligand,\, solvated\right),}$$

This equation can be used to determine the contribution of interaction in the complex and can be expressed as;

2 $$G= {E}_{Molecular\, Mechanics}- {G}_{solvated}- TS,$$

This equation can be further restructured to calculate the specific energy term.

3 $${\Delta G}_{bind}= {\Delta E}_{Molecular\, Mechanics}+ {\Delta G}_{solvated}- \Delta TS= {\Delta G}_{vaccum} + {\Delta G}_{solvated} ,$$

4 $${\Delta E}_{Molecular\, Mechanics}= {\Delta E}_{int}+ {\Delta E}_{electrostatic} + {\Delta E}_{vdW},$$

5 $$\Delta {G}_{solvated}= \Delta {G}_{Generalized\, born}+\Delta {G}_{surface\, area},$$

6 $$\Delta {G}_{surface\, area}= \gamma .SASA+b,$$

7 $$\Delta {G}_{vaccum}= {\Delta E}_{Molecular\, Mechanics}-T\Delta S.$$

The total binding energy is determined by the contribution of each of the terms mentioned above. Specifically, the free energy of ligand–protein/protein–protein or protein/nucleic acid total binding is represented by *ΔG*_*bind*_. The total gas phase energy, which is the sum of *ΔE*_*internal*_, *ΔE*_*electrostatic*_, and *ΔE*_*vdw*_*,* is reflected in *ΔE*_*MM*_. The sum of polar (*ΔG*_*PB/GB*_) and non-polar (*ΔG*_*SA*_) contributions to solvation is represented by *ΔG*_*sol*_. The conformational binding entropy, typically calculated through normal-mode analysis, is represented by *− TΔS*. The internal energy arising from various bonds, angles, and dihedral in molecular mechanics (MM) force field is reflected in *ΔE*_*internal*_ (in the MM/PBSA and MM/GBSA, this value is always zero as seen in the single trajectory of a complex calculation). *ΔE*_*electrostatic*_ and *ΔE*_*vdw*_ are the electrostatic and van der Waals energies calculated using MM, while ΔG_PB/GB_ represents the polar contribution to the solvation-free energy, calculated using Poisson–Boltzmann (PB) or generalized Born (GB) methods. *ΔG*_*SA*_ is the nonpolar solvation-free energy, usually calculated using a linear function of the solvent-accessible surface area (SASA)^44^. The conformational entropy was not calculated as it is computationally expensive and subjected to more inaccuracies.

#### Pan assay interference

During the early stages of drug design, it is crucial to screen for compounds that possess the desired pharmacokinetic properties, such as ADMET. To ensure the selection of high-quality compounds, an electronic filter known as Pan Assay Interference Compounds (PAINS) is employed, which focuses on identifying compounds of superior quality in databases^45^. The PAINS filter is designed to scrutinize compounds that have a higher probability of interfering with assays, displaying chemical reactivity, being frequently hit, and not being recognized by toxicophoric filters. To predict the ADMET characteristics of the compounds, we utilized an online PAINS server (https://biosig.lab.uq.edu.au/pkcsm/prediction). Those substances that passed the PAINS filter and exhibited superior ADMET characteristics.

## Results and discussion

In the current study pharmacophore based virtual screening, molecular modeling, and all-atoms simulation approaches have been utilized computationally to design potential small molecular drug for breast cancer. Breast cancer, the most common malignant tumor in women, involves progesterone and its receptor (HPR) in tumorigenesis and treatment sensitivity. We employed virtual screening and simulation methods to investigate the binding of final hits with HPR and further validated by using binding free energy approach. Through molecular docking and simulations, we gained valuable insights into the interactions between the selected lead compounds and the active site of HPR, identifying critical amino acid residues involved in ligand-receptor binding. The docking results demonstrated that the TCM38057/HPR exhibit the best docking energy of − 9.76 kcal/mol followed by TCM30460/HPR with the docking score of − 9.65 kcal/mol. Moreover, the docking results for ZINC32957366/HPR exhibit − 9.54 kcal/mol and ZINC57487561/HPR complexes revealed to be − 9.37 kcal/mol respectively. This structural understanding of HPR inhibition lays the groundwork for the rational design and optimization of novel HPR inhibitors for breast cancer therapy. The use of natural product-based compounds in this study holds significant promise for breast cancer treatment. Natural products are known for their diverse chemical structures, which may offer unique advantages over conventional synthetic drugs. Additionally, natural product-based inhibitors may present reduced side effects, making them attractive candidates for therapeutic development. The observed interactions and binding affinities of the lead compounds indicate their potential as viable alternatives agents to existing endocrine therapies targeting ER and PgR. Overall, this study contributes valuable insights into the role of HPR inhibition in breast cancer treatment and highlights the potential of natural product-based compounds as a viable therapeutic strategy. The integration of computational methodologies and molecular simulations not only provides a robust platform for identifying lead compounds but also accelerates the drug discovery process. Nonetheless, further experimental validation and preclinical studies are warranted to establish the efficacy and safety of these lead compounds in breast cancer therapy, ultimately advancing the field of breast cancer research and treatment.

### Pharmacophore based virtual screening

**Figure 2:**
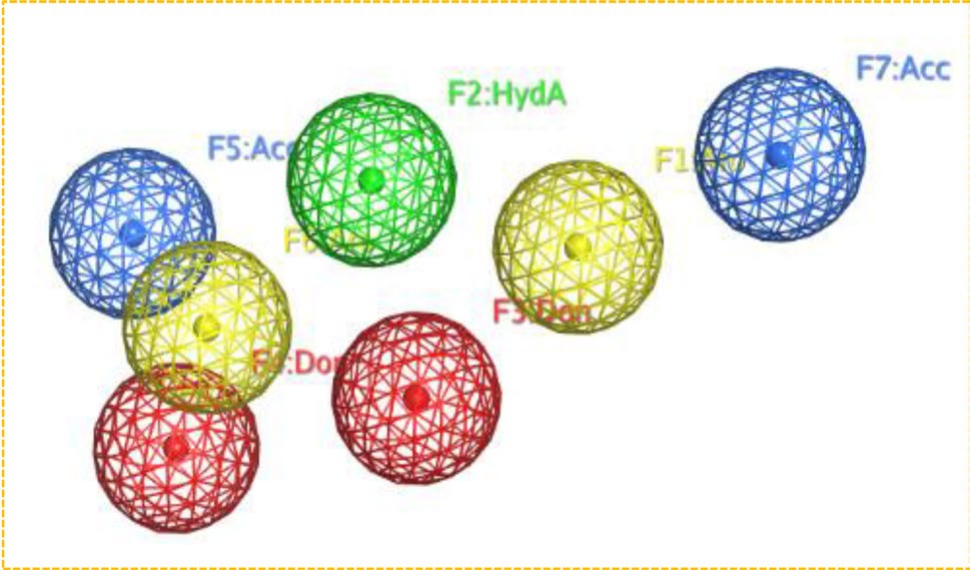
3-D pharmacophores features of the reference ligand containing total seven features. (PyMol v1.7, https://pymol.org/2/; Pro Lab v2018; https://originpro.informer.com/8.5/).

Pharmacophore modeling is a computational technique used in drug design and discovery to identify the essential features of a ligand that are critical for its interaction with a biological target (such as a protein). The underlying principle of this approach is to schematically represent the essential components of molecular recognition, enabling the identification and differentiation of molecules with similar biological activity and interactions with the target protein. Pharmacophore models describe the 3D arrangement of functional groups crucial for biological interactions with protein active sites. In ligand-based modeling, the prediction is made that similar compounds will exhibit comparable biological activity and bind to the target protein. This prediction is possible because the pharmacophore model emphasizes the critical features of the molecule involved in interactions and binding, rather than its overall structure. Ligand-based pharmacophore modeling is commonly employed to discover novel and potent ligands/inhibitors by comparing their molecular similarity to known promising inhibitors, without relying on protein structure information, which confers a significant advantage to this technique^46^. In this case, the pharmacophore model for the ligand (progesterone) binding in the protein structure consists of seven features: F1-arro (aromatic), F2-ess and HydA (essential and hydrogen bond acceptor), F3-Don (hydrogen bond donor), F4-ess and don (essential and hydrogen bond donor), F5-ess and Acc (essential and hydrogen bond acceptor), F6-arro (aromatic), and F7-Acc (hydrogen bond acceptor) Fig. 2. The pharmacophore model was validated by testing against a database of anti-cancer drugs, including progesterone as a reference. Then we used the model can be used to screen chemical databases (ZINC & TCM) for potential new compounds with similar features, aiding in the design and optimization of ligands with improved binding affinity and selectivity for the target protein. The pharmacophore model underwent validation by being tested against a collection of anti-cancer drugs, with Progesterone as a reference compound. Subsequently, this validated model was utilized to screen compounds sourced from the ZINC and TCM libraries. As a result of this screening, a multitude of potent compounds with robust interactions with the target protein were identified. 1450 Out of 100,000 compounds from ZINC, and 642 out of 40,000 compounds from the TCM database, structurally diverse hits were retrieved that match the Extended Huckel Theory (EHT) pharmacophore model. Finally, after applying Lipinski's Rule of Five (Ro5) for predicting oral bioavailability, 345 hits from the ZINC library and 137 hits from the TCM library were selected. These selected hits are likely to have better potential for oral absorption and may be promising candidates for further drug development.

### Molecular docking study of lead compounds

**Figure 3:**
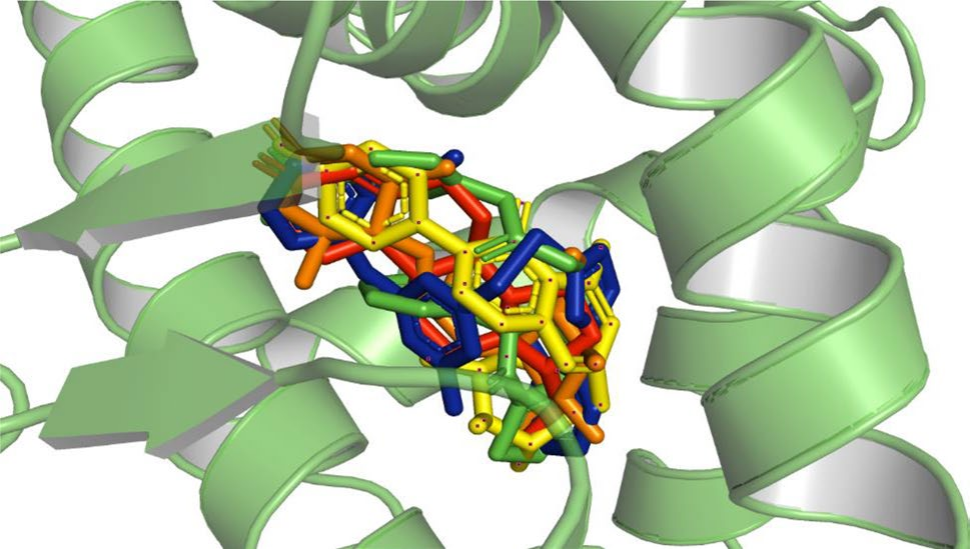
An illustration of the molecular surface of the human progesterone receptor (HPR) with all active hits superimposed, including the reference compound in red within the binding pocket. The ZINC57487561, ZINC32957366, TCM38057, TCM32702 and TCM30460 active ligands were represented by Green, Blue, Yellow, and Orange colors, respectively (PyMol v1.7, https://pymol.org/2/; Pro Lab v2018; https://originpro.informer.com/8.5/).

The docking results were then compared with the previously generated pharmacophore model (EHT pharmacophore model) to identify compounds that not only demonstrated favorable oral bioavailability but also exhibited key interactions with the essential pharmacophores features. This combined approach allowed for the prioritization of hits that possess both desirable pharmacokinetic properties and the ability to interact with the target protein in a manner consistent with the pharmacophore hypothesis. Consequently, a subset of compounds with strong docking scores and alignment with the pharmacophore model was identified, indicating their potential as promising candidates for further drug development. The potential anti-cancer compounds that were designed were seen to fit well within the human progesterone receptor drug target as shown in Fig. 3. Based on the combined results of Lipinski's Rule of Five (Ro5) filtering, molecular docking, and alignment with the EHT pharmacophore model, four compounds have been selected for further study, with two compounds originating from the ZINC library and two compounds from the TCM library. These findings synergistically support the rational design and discovery of new drug leads, increasing the likelihood of successful translation from virtual screening to experimental validation and potential clinical applications.

### Interaction details of final hits from TCM&ZINC datasets

**Figure 4:**
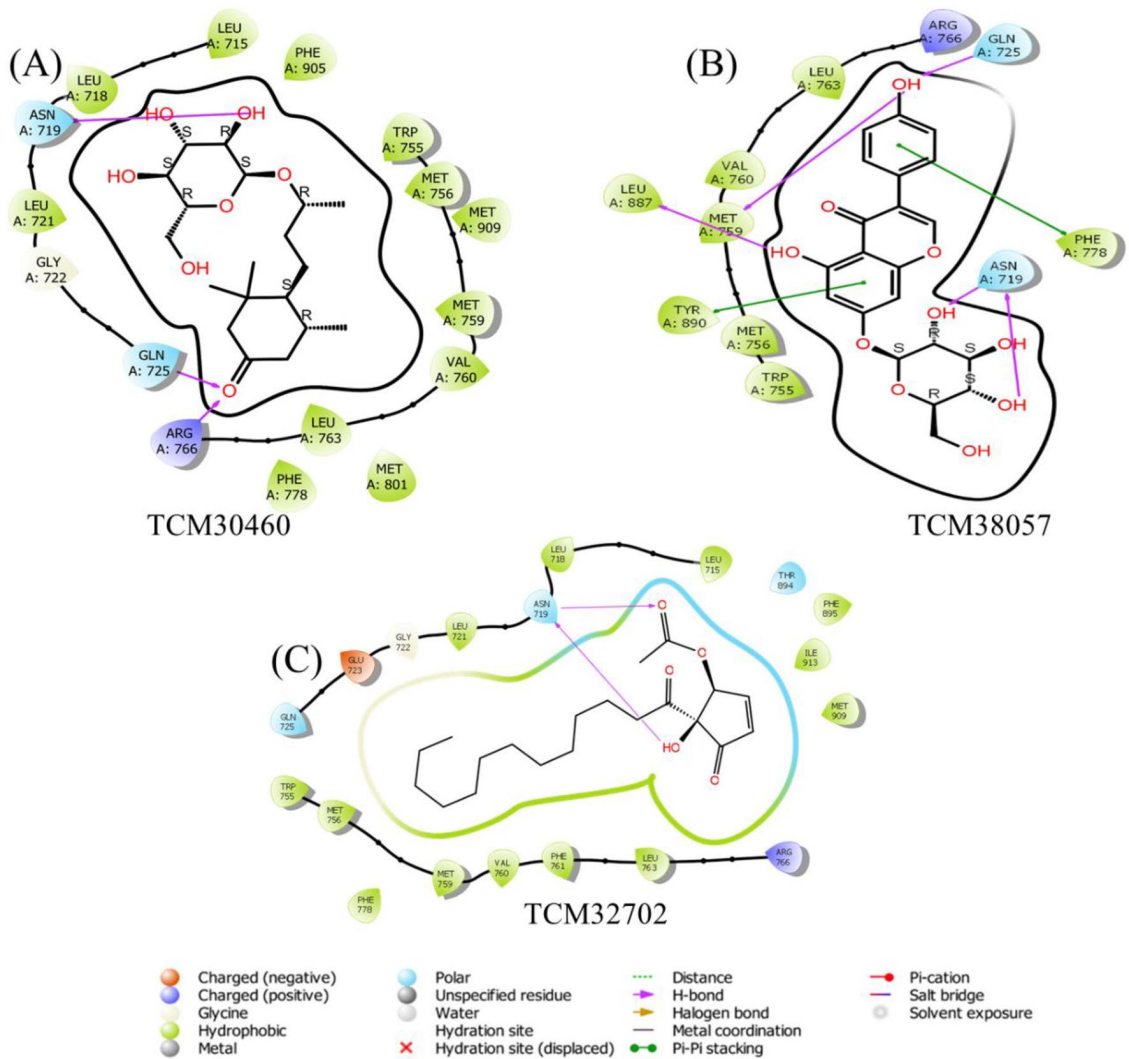
2-Dimensional binding interaction of selected hits from TCM database. (PyMol v1.7, https://pymol.org/2/; Pro Lab v2018; https://originpro.informer.com/8.5/).

**Figure 5:**
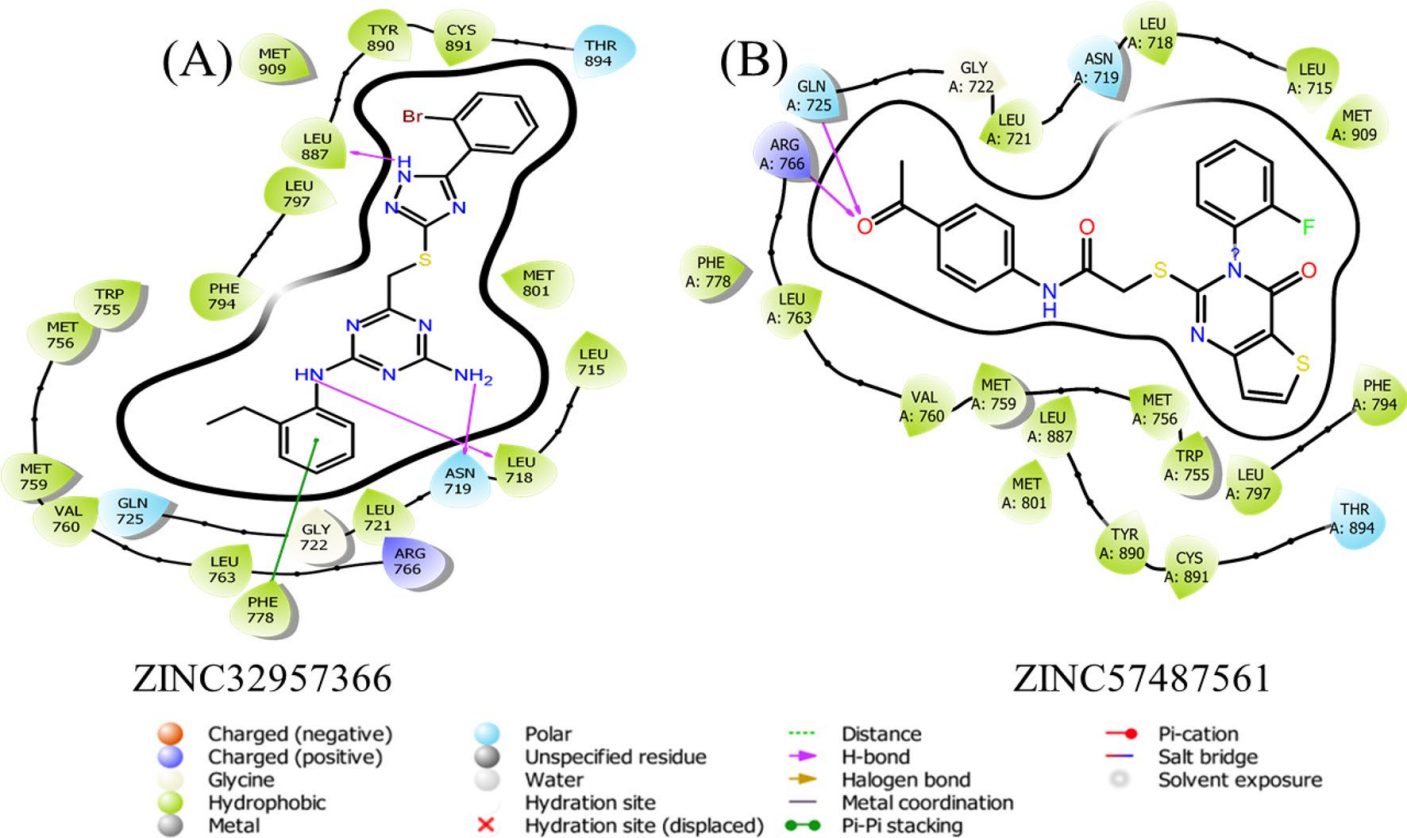
2-Dimensional binding interaction of selected hits from ZINC database. (PyMol v1.7, https://pymol.org/2/; Pro Lab v2018; https://originpro.informer.com/8.5/).

In order to understand the binding interaction information the 2D structure of the final hits compound was drawn on Maestro to investigate the potential binding interactions. The interaction results revealed promising binding interactions between the compound and the protein's active site. The compound was found to form key interactions, including hydrogen bonds, hydrophobic contacts, and electrostatic interactions. The docking analysis of TCM30460/HPR revealed the formation of three hydrogen bonds between the compound and residues Asn719, Gln735 and Arg766 with a bond distance 2.71 Å, 3.82 Å, and 4.00 Å respectively. These hydrogen bonds contribute to the stabilization of the compound within the active site and indicate potential key interactions for ligand binding Fig. 4a. Similarly, TCM38057/HPR demonstrated four hydrogen bond between the compound and residues, including Gln725, Met759, Leu887, and Asn719 with a bond distance 2.84 Å, 3.97 Å, 3.10 Å, and 2.80 Å. In addition, two Pi-Pi stacking with the residues Phe778, and Tyr890 respectively. These Pi-Pi stacking further enhance the binding affinity of the compound within the protein's pocket Fig. 4b. The interaction pattern of the TCM32702/HPR reported two hydrogen bonds which were also observed in the active site residues of HPR Fig. 4c. In case of ZINC32957366/HPR complex demonstrated three hydrogen bond with the residues Leu887, Leu718, and Asn719 with a bond distance 3.14 Å, 3.32 Å, and 2.95 Å. In addition, one Pi-Pi stacking with Phe778 Fig. 5. Similarly, ZINC57487561/HPR represents the total number of two hydrogen bond with the active site residues Gln725, and Arg766 with a bond distance 3.23 Å, and 2.90 Å Fig. 5. Overall, the docking analysis of the 2D structure drawn on Maestro provided insights into the potential binding interactions between the compound and the target protein. The observed hydrogen bonds, Pi-Pi stacking interactions highlight specific residues that are critical for binding. These findings serve as a starting point for further drug design studies against the breast cancer target. Table 1 represents docking result of the final hits are given below.

**Table 1:** 2D structures, database ID, toxicity, and docking scores of the top final five hits.

2D structures	TCM ID	Toxicity	Docking score kcal/mol
	TCM38057	None	− 9.76
	TCM30460	None	− 9.65
	TCM32702	None	− 9.23
	ZINC32957366	None	− 9.54
	ZINC57487561	None	− 9.37

### Protein structural stability analysis

**Figure 6:**
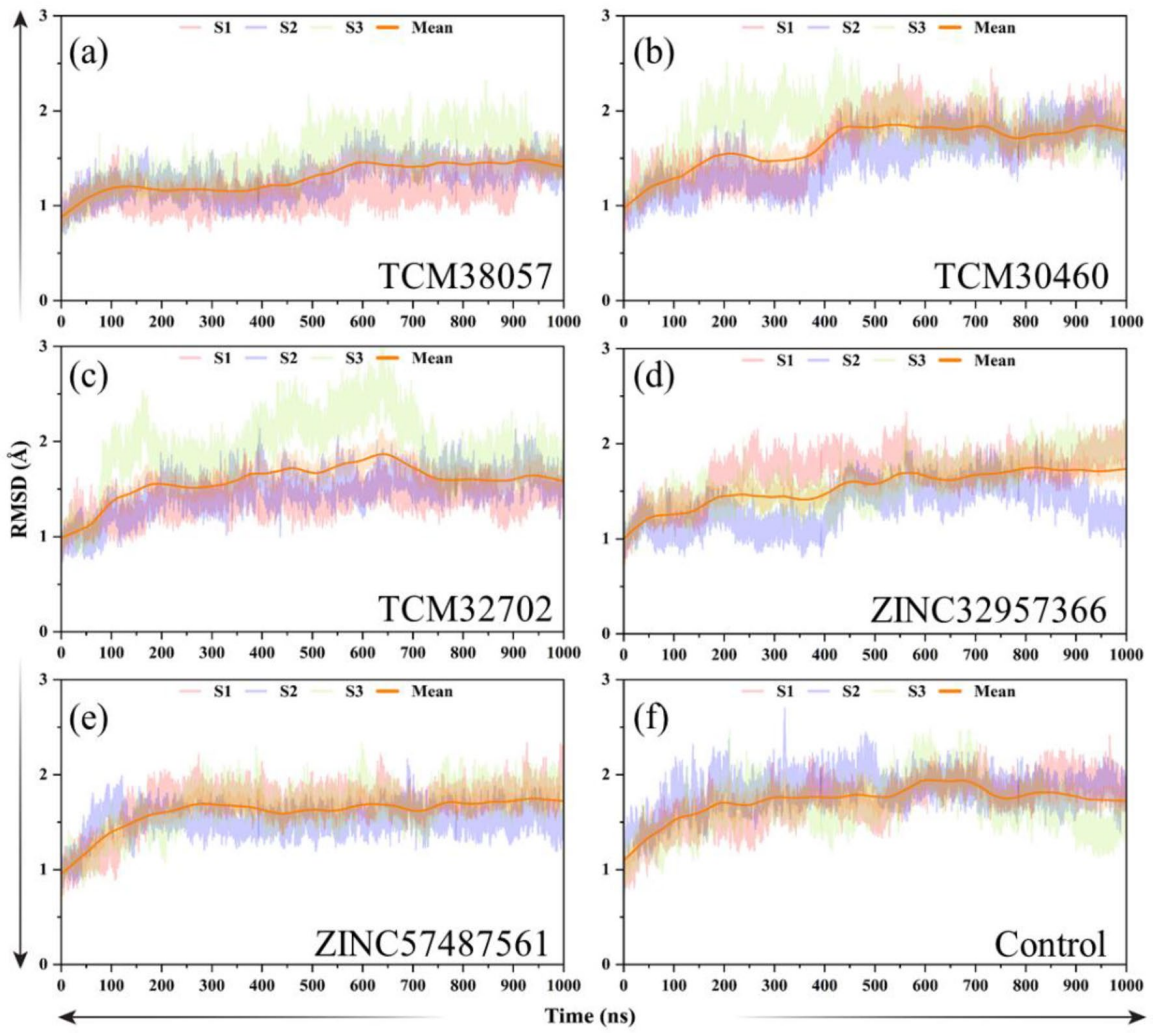
(**a**–**f**) Dynamic stability assessment of the control/HPR, and five top hits complexes.

Assessing the dynamic stability of a protein bound to a ligand is essential to demonstrate the pharmacological activity of that particular compound. The stabilities of the five selected complexes, along with the reference complex, were determined by calculating the root mean square deviation (RMSD) of the backbone atoms. The ZINC57487561/HPR reported stable dynamic behavior with no significant structural perturbation. At the beginning, the RMSD gradually increased over time. From 20 ns to the end, the RMSD graph maintains a stable trend without significant fluctuations, so the complex demonstrated stable dynamics with an average RMSD of 1.20 Å, because when RMSD ≤ 2 Å can prove the reliability of the docking method. In contrast, the ZINC32957366/HPR reported significant structural perturbation until 600 ns. The RMSD continues to increase with abrupt increase and decrease between 10 and 600 ns. And the maximum value appeared at 550 ns, with a maximum value of 1.70 Å. The complex demonstrated a uniform RMSD pattern from 20 ns to the end. Although there are fluctuations in the RMSD image but its average value is 1.4 Å, and a uniform straight RMSD was demonstrated and thus behave stably during the simulation. In general, ZINC32957366/HPR and ZINC57487561/HPR are relatively stable. The RMSD for the top hits from TCM database were also computed to reveal the stability profiles of these molecules. Among the top hits such as TCM30460/HPR and TCM38057/HPR complex were subjected to RMSD analysis. These three compounds i.e. TCM30460/HPR, TCM38057/HPR, and TCM32702/HPR demonstrated alike behavior with no significant perturbation during the simulation and stabilized at 1.30 Å each. And the RMSD graph of these three compounds showed an upward trend before 150 ns. The TCM38057/HPR demonstrated a uniform RMSD pattern between 600 and 1000 ns with the gradual increasing trend but then significant perturbations were seen in the complex at the end of the simulation, and the RMSD graph shows a downward trend. In contrast, The RMSD graph of TCM30460/HPR shows no significant fluctuations between 400 and 1000 ns. But the RMSD had large fluctuations and continues to increase with abrupt increase and decrease between 0 and 300 ns. Similarly, the significant perturbations were seen in the complex at the end of the simulation. In general, the average RMSD of these three compounds i.e. TCM30460/HPR, TCM32702/HPR and TCM38057/HPR are 1.30 Å, because when RMSD ≤ 2 Å can prove the reliability of the docking method (Fig. 6).

### Residual flexibility analysis

**Figure 7:**
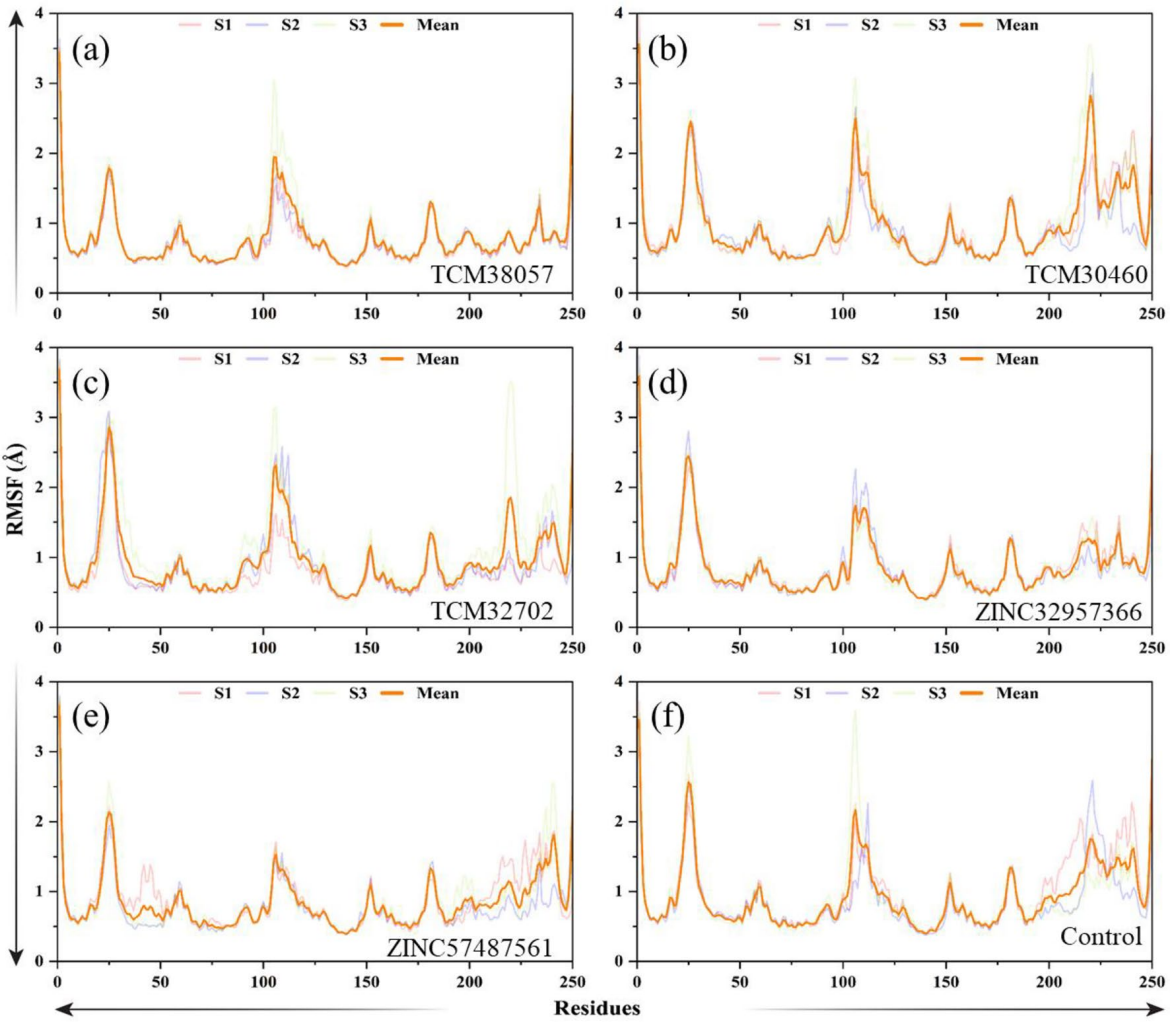
(**a**–**f**) Residual flexibility assessment of the control/HPR, and five top hits complexes.

In molecular dynamics (MD) simulations, the root mean square fluctuation (RMSF) is a useful metric and can be used to compare the flexibility of different regions within a molecule or between different molecules. This can help identify flexible regions that may be important for ligand binding or protein–protein interactions. RMSF is also an important parameter for validating MD simulations. Experimental measurements of RMSF can be used to validate the accuracy of the simulation and the force field used. Both the ZINC32957366 and ZINC57487561 presented higher flexibility are shown in Fig. 7. In case of ZINC compound ZINC32957366 demonstrated significantly higher residues flexibility in the regions 25–50 and 150–200. At the same time, it also maintains a certain level of flexibility in other regions. The RMSF graph of ZINC32957366 exhibits significant fluctuations but overall maintains high flexibility. In contrast, the RMSF graph of ZINC57487561 is relatively stable, demonstrated significant higher flexibility in residues within the regions 100–150 and 180–250. The minimum value of RMSF graph is higher than 6 Å. The RMSF graph of the TCM30460, TCM32702 and TCM38057 have similar trends, and their graph trajectories are similar. The RMSF value of TCM30460 is generally smaller than that of TCM38057, so the flexibility of TCM30460 is also smaller than that of TCM38057. The RMSF value of TCM38057 is generally high, because the minimum value of RMSF graph is higher than 6 Å. And TCM38057 demonstrated significantly higher residues flexibility in the regions 20–40, 75–150 and 170–200. Similar to TCM38057, the TCM30460 also demonstrated significantly higher residues flexibility in the regions 20–40, 75–150 and 170–200. However, in other regions, the RMSF value of TCM30460 is relatively low. Overall, TCM30460 is also have a high flexibility. In general, both the TCM30460 and TCM38057 have a certain level of flexibility, and the RMSF value of TCM38057 is generally more than that of TCM30460.

### Radius of gyration (Rg) analysis

**Figure 8:**
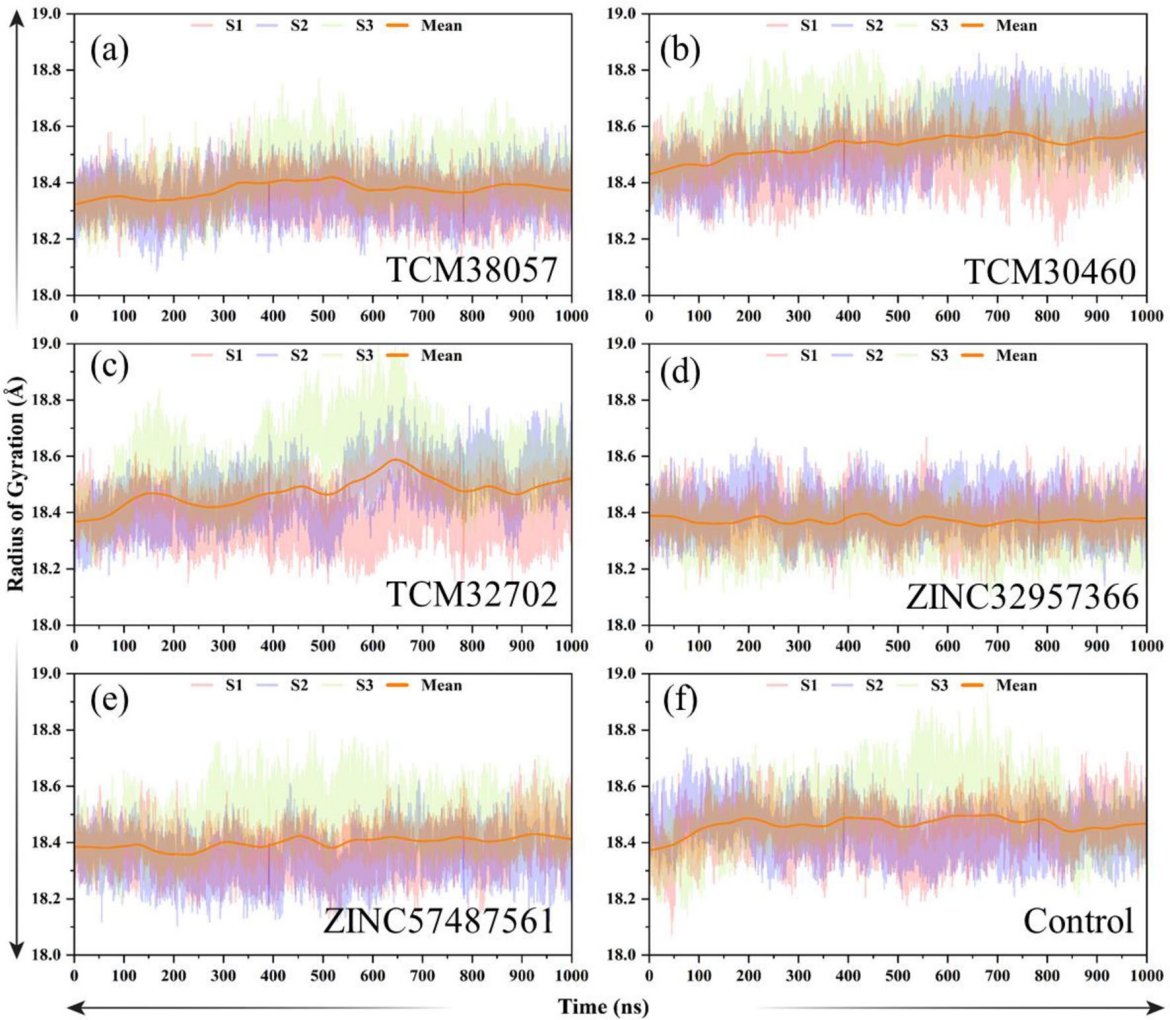
(**a**–**f**) Radius of gyration (Rg) of the control/HPR, and five top hits complexes.

The Radius of Gyration (Rg) analysis was performed to characterize the compactness and overall size of the protein throughout the simulation trajectory. The Rg values were calculated for each frame of the simulation. Figure 8 shows the Rg profile of the protein over time. The x-axis represents the simulation time (ns), while the y-axis represents the Rg values in angstroms. The average Rg values for ZINC32957366/HPR were recorded 18.2 Å with a little fluctuation throughout the all simulation. Similarly, ZINC57487561/HPR the average value was recorded 18.3 Å thus convey a stable binding of the ligand with no significantly increase or decrease in the protein size during simulation Fig. 8a. And the Rg value of the reference/HPR is not very stable, showing significant fluctuations. The Rg for the TCM30460/HPR complex started to decrease initially and reached 18.20 Å at 30 ns and then equilibrated. A straight uniform Rg was seen from 40 ns until the last simulation time. Overall initially, at the beginning of the simulation, the protein exhibited a relatively low Rg value, indicating a less extended conformation. Moreover, the Rg for TCM38057/HPR started to increase initially and reached to 18.4 Å, however after it decrease gradually and attained the tighter structural packing at 800 ns, which continues to follow the same pattern until 1000 ns Fig. 8b. These analyses revealed that the regions with a higher Rg value corresponded to more flexible and disordered regions, such as loops and coil regions. In contrast, regions with lower Rg values were associated with more structured elements, including alpha-helices and beta-sheets. These findings suggest that the protein undergoes conformational changes during the simulation, transitioning from a more extended state to a more compact and structured state. This compactness might be crucial for the protein's stability, folding, and interactions with ligands or other biomolecules.

### Hydrogen bond analysis

**Figure 9:**
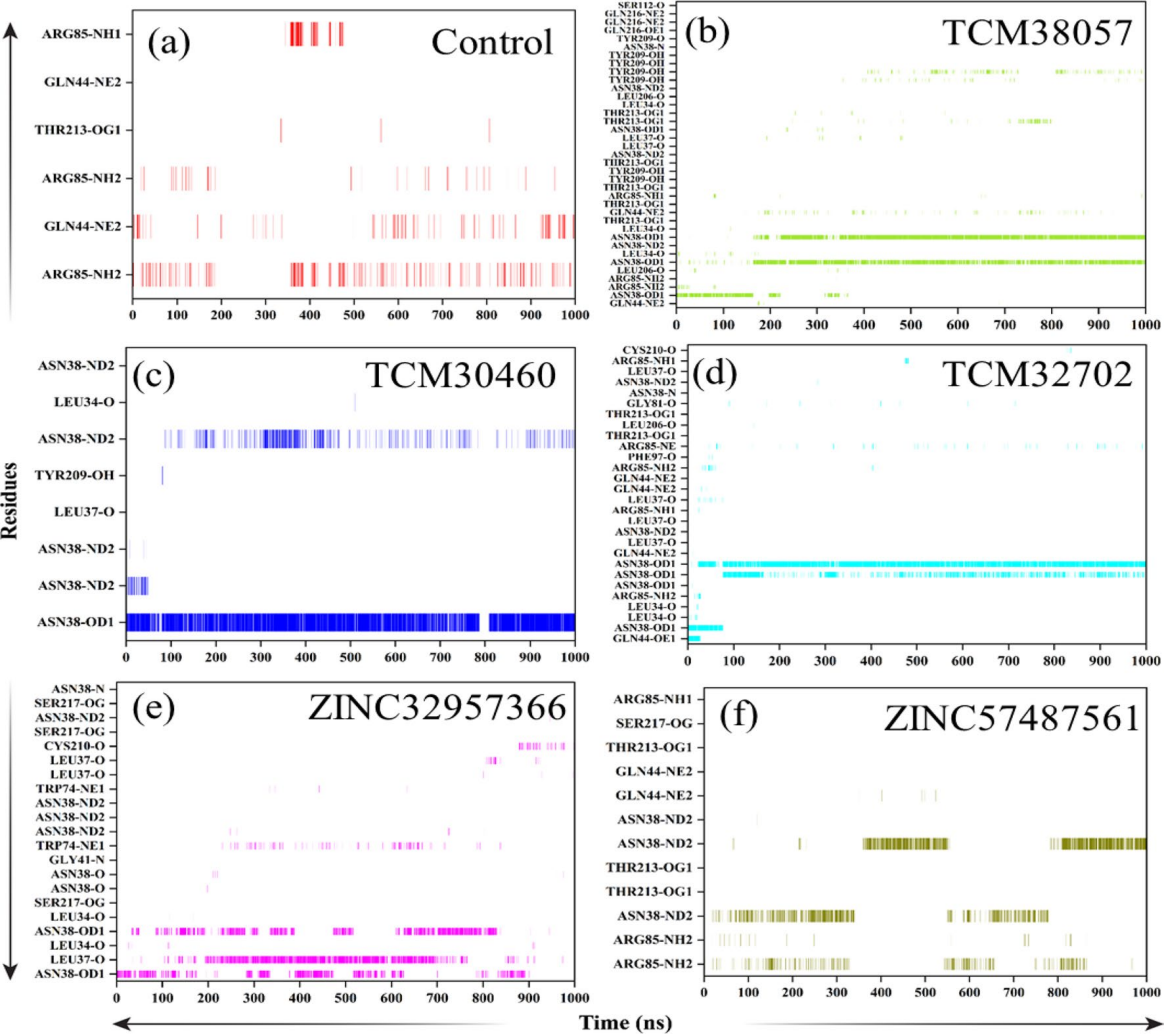
Hydrogen bonding analysis for the control and top five hits identified through pharmacophore based screening.

Hydrogen bond analysis was performed to identify potential hydrogen bonding interactions within the protein. Hydrogen bonds were calculated based on distance and angle criteria using a cutoff of 3.5 Å for the donor–acceptor distance and 30 degrees for the donor-hydrogen-acceptor angle. The analysis revealed the presence of several hydrogen bonds within the protein structure. Figure 9a–d shows a graphical representation of the hydrogen bond network. Each line represents a hydrogen bond, with the donor and acceptor residues labeled accordingly. Interestingly, residues involved in hydrogen bonding interactions were found to be consistent with the regions of high flexibility identified through the RMSF analysis. Residues 150–200 and 80–130, which exhibited higher RMSF values, also showed a higher propensity for forming hydrogen bonds with neighboring residues. The presence of hydrogen bonds in these flexible regions suggests their involvement in stabilizing local conformations and facilitating structural rearrangements. Additionally, the hydrogen bond network also extended to other regions of the protein, contributing to the overall stability and structural integrity. These combined findings from the RMSF and hydrogen bond analyses provide a comprehensive understanding of the dynamic behavior and intermolecular interactions within the protein structure. The identified flexible regions and hydrogen bond networks can guide further investigations into the protein's function, ligand binding, and potential drug targeting strategies.

### Binding free energy calculation

Molecular Mechanics/Generalized Born Surface Area (MM/GBSA) analysis was performed to assess the binding free energy and contribute to our understanding of the protein–ligand interactions. The MM/GBSA calculations were carried out for the protein–ligand complexes sampled during the molecular dynamics simulations. The binding free energy (ΔG_bind) is represented as the sum of the contributions from different terms, including the molecular mechanic's energy (ΔG_MM), the solvation energy (ΔG_GBSA), and the entropy contribution (ΔS) are shown in Table 2. The standard deviation (SD) of the calculated values is also provided to indicate the reliability of the results. The calculated ΔG_bind values indicate the overall binding affinity of the ligands to the protein. Negative ΔG_bind values suggest favorable binding, while positive values indicate unfavorable binding. In our study, ZINC32957366/HPR, ZINC57487561/HPR, TCM30460/HPR, and TCM38057/HPR displayed ΔG_bind values of − 67.6065 ± 2.6532 kcal/mol, − 60.3815 ± 2.6222 kcal/mol, − 46.1585 ± 2.4538 kcal/mol, and − 48.3541 ± 2.6340 kcal/mol respectively, indicating strong binding affinities. Overall, the MM/GBSA analysis provided valuable insights into the binding free energy and the key molecular interactions between the protein and ligands. These findings enhance our understanding of the structure–activity relationships and guide future drug design efforts targeting the protein of interest.

**Table 2:** Binding free energy results calculation using MM/GBSA approach.

Parameters	ZINC32957366/HPR	ZINC57487561/HPR	TCM30460/HPR	TCM38057/HPR	TCM32702/HPR	Control/HPR
ΔTotal	− 67.60 ± 2.65	− 60.38 ± 2.62	− 46.15 ± 2.45	− 48.35 ± 2.63	− 44.21 ± 1.10	− 40.23 ± 2.09
ΔEvdw	− 69.99 ± 2.64	− 64.82 ± 2.55	− 50.87 ± 2.30	− 48.35 ± 2.63	− 44.83 ± 2.17	− 49.69 ± 2.10
ESURF	− 7.43 ± 0.15	− 7.03 ± 0.13	− 6.72 ± 0.15	− 7.21 ± 0.19	− 3.42 ± 0.51	− 5.62 ± 0.51

#### The pan assay interference compounds (PAINS) filter assay

PAINS electronically filters quality compounds in the database targeting substances that could potentially disrupt assays due to their higher chemical reactivity, leading to a higher incidence of false positive hits. All the four compounds from both database were passed from the electronic filter as well as their ADMET properties were studied using online pain server Table 3. During drug design, it is always recommended to subject compounds to multiple filtrations for desirable pharmacokinetic features, such as ADMET characteristics.

**Table 3:** The compounds that passed the filter with appropriate drug like properties.

Compound ID	Pains filter	2D structure	IUPAC name
TCM38057	Pass		Acetic acid 1-[2-(3-cyclopentyloxy-4-hydroxy-phentyl)-ethyl]-3,10-dihydroxy-decyl ester
TCM30460	Pass		5-(1-5[5-Butyl-furan-2-yl)-ethyl]-2-hydroxy-phenoxymethyl)-cyclohexyl-3-isopropyl-3H-pyrrolium
TCM32702	Pass		Acetic acid 2-hydroxy-3-oxo-2-tridecanoyl-cyclopentyl ester
ZINC32957366	Pass		2-Amino-6-[4-(1,3-dicarboxy-propylcarbamoyl)-phenylamono]-methyl-4-oxo-3,4,5,6,7,8-hexahydro-pterdin-1-ium
ZINC57487561	Pass		1-[5-methyl-2-phenyl-3-(1,2,4-triazol-1-yl)hex-1-enyl]-1,2,4-triazole

## Conclusion

Summarily, this study attempted to search for potential inhibitors of Human progesterone receptor using pharmacophore based virtual screening, and MD simulation techniques. To this end, the crystal structure of HPR were retrieved from protein databank along with the reference ligand (progesterone) and subjected to a screening against ZINC and TCM datasets aimed at determining their binding potentials to the HPR and assessing their potential to serve as drugs based on following Ro5. The results derived from the screening pipeline revealed ZINC32957366, ZINC57487561, TCM30460, TCM32702 and TCM38057 as the most viable compounds that are worthy of exploration in future efforts. Ultimately, this study could serve to provide a scientific basis for the exploration of novel therapeutic modalities for combating breast cancer therapy, upon further experimental validation.

## Data Availability

All data generated or analyzed during this study are included in the article.
